# Evaluation of a Video-Based Concept for Hand Hygiene Education of Parents in a Neonatal Intensive Care Unit

**DOI:** 10.3390/healthcare12171766

**Published:** 2024-09-04

**Authors:** Judith Rittenschober-Böhm, Johanna Strassl, Maria Kletecka-Pulker, Péter Szerémy, Tamás Haidegger, Tamás Ferenci, Angelika Berger, Michael Wagner

**Affiliations:** 1 Division of Neonatology, Pediatric Intensive Care and Neuropediatrics, Department of Pediatrics and Adolescent Medicine, Comprehensive Centre for Pediatrics, Medical University of Vienna, 1090 Vienna, Austriaangelika.berger@meduniwien.ac.at (A.B.); michael.b.wagner@meduniwien.ac.at (M.W.); 2 Ludwig Boltzmann Institute Digital Health and Patient Safety, Medical University of Vienna, 1090 Vienna, Austria; maria.kletecka-pulker@univie.ac.at; 3 Institute for Ethics and Law in Medicine, University of Vienna, 1010 Vienna, Austria; 4 HandInScan Zrt, 4025 Debrecen, Hungary; peter.szeremy@irob.uni-obuda.hu (P.S.); haidegger@irob.uni-obuda.hu (T.H.); 5 University Research and Innovation Centre (EKIK), Óbuda University, 1034 Budapest, Hungary; 6 John von Neumann Faculty of Informatics, Óbuda University, 1034 Budapest, Hungary; ferenci.tamas@nik.uni-obuda.hu

**Keywords:** hand hygiene, infection control, neonatal intensive care, patient safety, preterm birth

## Abstract

Background: Current clinical guidelines support family-centered care in Neonatal Intensive Care Units (NICUs). This implies parents should also be involved in the most critical patient safety measures. Hand hygiene is the single most important tool to prevent healthcare-associated infections and related long-term effects. Although often studied in healthcare workers, the hand hygiene compliance of parents is rarely assessed. The aim of this study was to evaluate the effectiveness of an educational video, available in ten different languages, in teaching parents hand hygiene techniques in a NICU, lowering the burden on the staff. Methods: Parents in the intervention group were educated through a video; the control group received personal instruction from healthcare workers. The primary outcome parameter was the predicted probability of passing a subsequent hand scan. Results: The quality of hand hygiene among parents educated through the video was at least as good as that of those who received instruction from a healthcare worker, demonstrated by a higher predicted probability of passing the hand scan (43.8% vs. 57.1% in male and 67.9% vs. 75.9% in female participants). The feedback from the intervention group was predominantly positive, with most parents (62%) expressing a preference for video-based education. Conclusion: Implementing a video-based approach seems to be effective for educating parents about hand hygiene in a NICU and was well accepted by the parents. This method offers a consistent standard of hand hygiene education, helps to overcome language barriers, and can also be used as regular reminder of the importance and proper technique of hand hygiene.

## 1. Introduction

Healthcare-associated infections (HAIs) constitute a major problem in intensive care units and represent challenges for patient safety. Newborns and preterm-born infants are especially vulnerable due to their immature immune systems and skin and frequent invasive procedures [1,2]. HAIs are significant risk factors for morbidity and mortality as well as adverse neurodevelopmental outcomes in preterm-born infants [3,4]. Moreover, the use of antibiotics in early childhood has been associated with long-term adverse effects such as childhood allergies, asthma, childhood overweight, and obesity [5,6]. Finally, HAIs also represent a relevant economic factor as each episode of sepsis in preterm infants increases the duration of stay in hospital and the costs [1].

Hand hygiene (HH) is the single most important tool to prevent HAIs [7]. Organisms causing HAIs are often transmitted via the hands of physicians, nurses, and other caregivers attending to the infants [8]. Consequently, improving HH continues to be a fundamental goal in preventing HAIs and their associated long-term consequences [1].

Patient empowerment has been a widely employed and celebrated method to improve the efficacy of infection prevention measures in clinical settings [9]. In the Neonatal Intensive Care Unit (NICU), integrating parents into the care process is of paramount importance [10]. Early skin-to-skin contact and kangaroo care have been shown to significantly affect both the short- and long-term outcomes of preterm infants. Initiating these practices as soon as possible after birth is crucial [11,12,13]. This makes them part of the complete patient safety net; therefore, their participation in infection prevention training and practice has a comparable importance to that of the healthcare workers (HCWs) working at the department.

As part of the family-friendly NICU and parent empowerment concepts, it is imperative that parents also adhere to optimal HH practices. While several studies have focused on the HH compliance of nurses and physicians [14,15,16], other caregivers such as parents are often neglected. Parents’ HH compliance and performance has been identified as rather low, which needs to be improved [17,18,19]. However, acknowledging the challenges in daily clinical practice is crucial. Teaching parents to adhere to the best HH practice can be very time-consuming for HCWs and may be further complicated by language barriers. In a study performed by Kletecka-Pulker et al. on language barriers in public health in Vienna, it was found that 71% of HCWs encountered language barriers at least two to three times a week. Moreover, 81% of HCWs reported relying on relatives or other non-professional interpreters for translation [20]. This underscores the significant challenge of language barriers present in ensuring adherence to best HH practices among parents in clinical settings. Moreover, since education in HH for parents should start immediately after a baby is admitted to the NICU, it is often provided by different HCWs. As a result, the education provided is likely heterogeneous.

In their comprehensive review on HH perspectives, Pittet et al. outlined a “hand hygiene research agenda for 2021 and beyond” [21,22]. One of the included points was the critical need for studies focusing on enhancing participation through identifying the most effective methods for HH education.

One device focusing on hand hygiene education is the Semmelweis System (Handinscan Zrt. Debrecen, Hungary), a CE-marked training tool designed to educate users on proper hand disinfection techniques by providing visual feedback on appropriately versus inappropriately disinfected areas of the hand, as earlier validated and described [15,23,24]. The device generates a digital-image-based evaluation of the hand surface coverage using an ultraviolet-dye-labeled alcohol-based disinfectant and can objectively quantify the extent of hand coverage. Artificial intelligence-based image processing identifies missed areas and calculates the percentage of the hand surface coverage.

The aim of this study was to test the hypothesis that an educational video available in ten different languages is as effective as personal instruction from an HCW in teaching parents in a NICU proper HH techniques, as assessed by the Semmelweis System, and to evaluate whether this approach is well accepted by the parents.

## 2. Materials and Methods

### 2.1. Study Design

**Figure 1:**
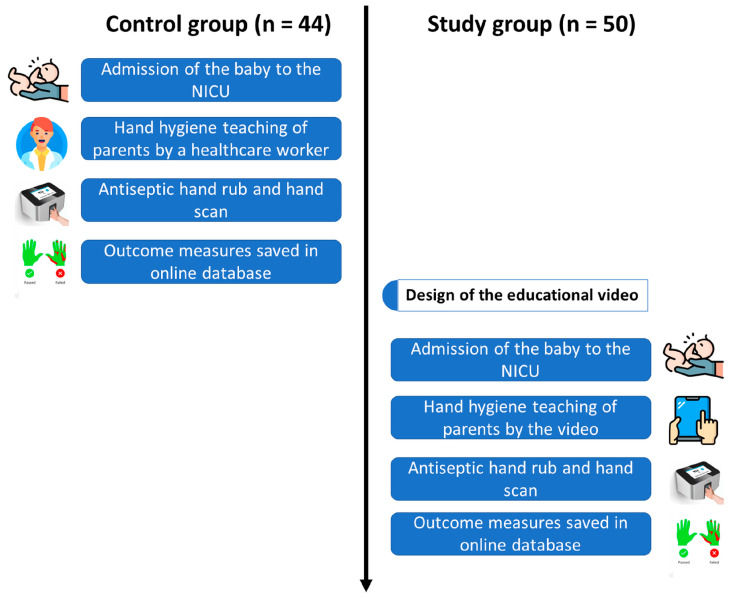
Study flow chart. After their baby’s admission to the NICU, parents in the control group received conventional education from an HCW, followed by a hand scan using the Semmelweis System. Starting with the implementation of the educational video, parents in the intervention group were educated via the video before undergoing a hand scan as well.

In order to evaluate the effectiveness of a video-based concept in educating parents on HH, we conducted an interventional study and analyzed the quantified hand antisepsis technique based on the HH education group. Parents in the intervention group were educated by a video; the control group received personal instruction from an HCW (Figure 1).

A four-minute animated video, addressing the importance and technique of hand antisepsis, was designed by the Division of Neonatology of the Medical University Vienna, in cooperation with the Institute for Ethics and Law in Medicine, University of Vienna, and produced by AniMedical KG, Linz, Austria. The video was translated into 9 further languages by SAVD Videodolmetsch GmbH, Vienna, Austria, and is available in English, German, Arabian, Bosnian, Croatian, Farsi, Kurdish, Russian, Serbian, and Turkish. The video is freely available online in German [25].

### 2.2. Setting and Participants

**Figure 2:**
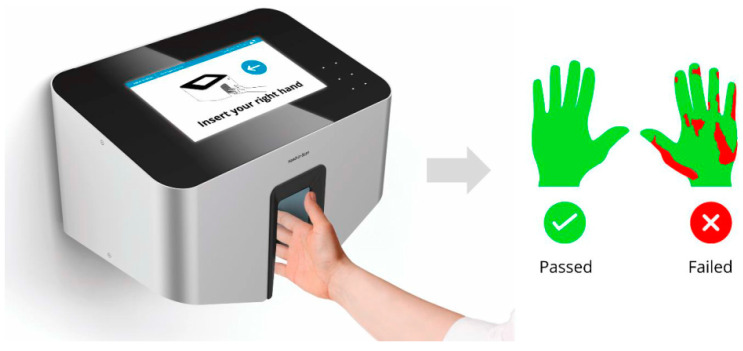
Semmelweis System. The digital health tool is able to assess the coverage of ultraviolet-dye-labeled alcohol-based handrub on hands after regular hand hygiene (Image: courtesy of HandInScan Zrt).

This study was performed in the level III NICU of the University Hospital in Vienna, Austria, over a study period of 6 month. As part of standard procedure, parents receive hand hygiene education ideally within 24 h of their baby’s admission to the NICU. All parents of newly admitted babies were eligible to participate in the study as long as there was no reason preventing them from performing antiseptic HH. When parents arrived for hand hygiene education, a member of the study team invited them to voluntarily participate in this study and, upon agreement, assigned them to the intervention group. Consequently, they were provided with a tablet (iPad, Apple, Cupertion, CA, USA) that offered the educational video available in 10 different languages. The parents were seated in the parents’ waiting room and had the freedom to select their preferred language. It was left to their discretion how many times they wished to watch the video. In the event that both parents of one baby agreed to take part in the study, they watched the video separately to avoid bias regarding language and how often they wanted to watch the video. After watching the video, parents were asked to perform a hand rub with an ultraviolet-dye-labeled alcohol-based disinfectant (Optik, Schülke & Mayr GmbH, Norderstedt, Germany) followed by a hand scan using the Semmelweis System to assess the coverage of their hands’ surface with the alcohol-based hand rub and to give objective feedback on HH quality (Figure 2). The control group consisted of parents who performed a hand rub and hand scan after conventional face-to-face HH education by an HCW in an earlier study conducted on the same NICU before the implementation of the video (unpublished data). Personal data of participants regarding sex, age, dominant hand, and earlier HH education were collected. Moreover, parents in the intervention group received a questionnaire regarding their attitude and experiences related to HH and the educational video. All parents were invited to use the scanner for self-check on a regular basis.

### 2.3. Outcome Measures

To assess the HH technique and provide objective feedback, the Semmelweis System (HandInScan Zrt., Debrecen, Hungary) was used (Figure 2) [23]. Results were saved in an online reporting database for evaluation. The primary outcome parameter was the predicted probability (PP) of passing the hand scan, based on the HH education group. Using established educational metrics, a minimum coverage of 95% of each hand surface (including the palm and dorsum of both the left and right hands) was considered adequate for parents. This level of coverage was categorized as “passed” [26]. If the coverage of any of the four surfaces was below 95%, the whole measurement was categorized as “failed”. The secondary outcome parameter was the identification of the most frequently missed areas of hand antisepsis. Each hand surface was divided into 12 regions), and a lack >0.5 cm^2^ in coverage in any of these particular regions was counted as a miss, which was later computed for all measurements.

Moreover, parents’ acceptability of the video was evaluated.

### 2.4. Statistical Analysis

Hand antisepsis was computed as a percentage of the measured coverage with ultraviolet-dye-labeled alcohol-based disinfectant. Passing of the test (defined as a result > 95%) was modeled as a binary variable with multivariable logistic regression using sex, number of video watches (categorized as 1, 2, ≥3), and earlier hand hygiene education when modeling within the study group and group and sex when modeling both groups. In the latter case, interaction was allowed between group and sex. The results are visualized as predicted probability of passing the test with 95% confidence intervals. With respect to the most frequently missed areas, the average occurrence of failure in each region was computed for all measurements.

Calculations were carried out under the R statistical program package version 4.3.0 (R Foundation for Statistical Computing, Vienna, Austria).

## 3. Results

### 3.1. Participants

A total of 94 parents participated in the study, 44 in the control group and 50 in the intervention group. Demographic data are shown Table 1. Among the 50 participants of the intervention group, nine first languages were represented: 31 spoke German, 3 Farsi, 3 Slovak, 3 Serbian, 2 Croatian, 2 Polish, 2 Romanian, 1 Albanian, and 1 Turkish. The video was available in five of these languages. In total, 7 participants opted to watch the video in a language other than German: (3 in Arabic, 2 in English, 1 in Serbian, and 1 in Turkish). Two participants did not provide information on their first language or the chosen language for the video on the questionnaire.

### 3.2. Hand Scan Performance

The hand antisepsis technique of parents after HH education was evaluated by calculating the predicted probability (PP) of passing the hand scan, depending on the HH education group.

**Figure 3:**
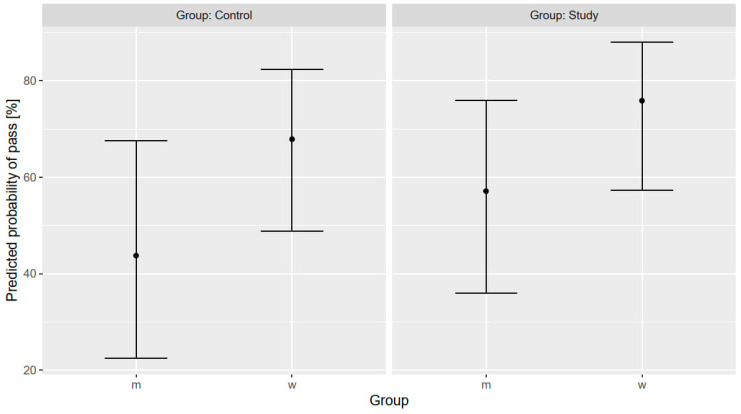
Hand scan performance. Predicted probability of passing the hand scan with at least 95% coverage: (**left**) control group, educated by an HCW; (**right**) intervention group, educated by the video; m, male parents; w, female parents;.

Figure 3 displays the PP of achieving a minimum coverage of 95% on hand scans, categorized by the type of HH education received and by sex. In both groups, women had a higher predicted probability of passing the scan compared to men. For female participants, the PP of passing the hand scan was 67.9% (95% CI 48.9–82.4%) in the control group compared to 75.9% (95% CI 57.3–88.0%) in the intervention group. In contrast, male participants demonstrated a significantly lower PP of passing, with a more pronounced difference between the groups: 43.8% (95% CI 22.5–67.6%) in the control group versus 57.1% (95% CI 36.0–76.0%) in the intervention group.

**Figure 4:**
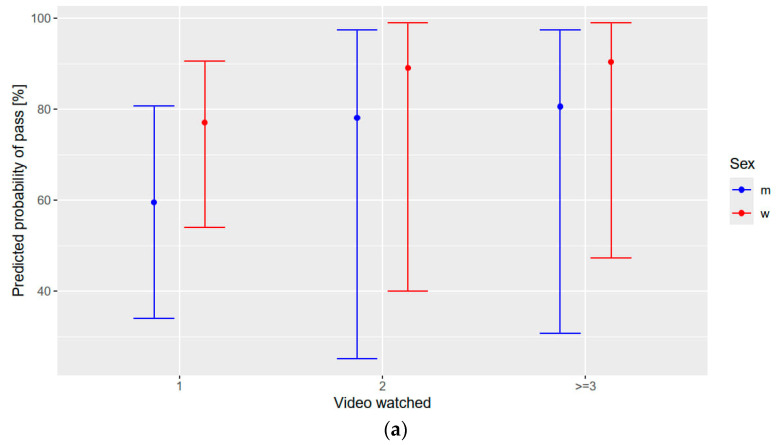

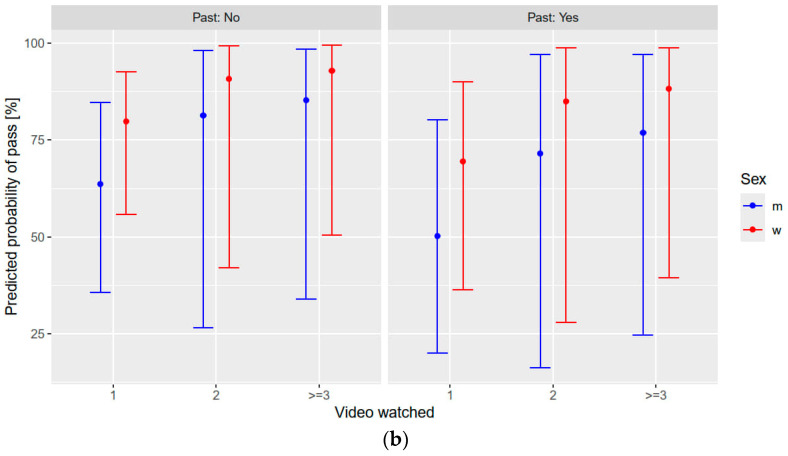
(**a**) Hand scan performance and number of video views. Predicted probability of the intervention group passing the hand scan with at least 95% coverage, depending on the number of times they watched the video: (**left**) one time; (**middle**), two times; (**right**) three or more times. m, male parents; w, female parents. (**b**) Hand scan performance, number of video views, and former hand hygiene education. Predicted probability of the intervention group passing the hand scan with at least 95% coverage, depending on the number of times they watched the video and former hand hygiene education: (**left**) no former hand hygiene education; (**right**) former hand hygiene education. m, male parents; w, female parents.

Figure 4a shows the PP of passing the hand scan in the intervention group, based on the number how often participants watched the video. For male participants, the PP increased from 59.5% (95% CI = 34–80.8%) to 78,1% (95% CI = 25.1–97.4%) when the video was watched twice compared to just once. Similarly, for female participants, the PP rose from 77.1% (95% CI = 54–90.6%) to 89,1% (95% CI = 40.0–99.0%) under the same conditions. However, the increase in PP when watching the video three or more times was relatively modest, reaching 80,6% (95% CI = 30.7–97.5%) for male participants and 90.5% (95% CI = 47.3–99.1%) for females. On average, the participants watched the video 1.65 times (range: 1–10 times).

Figure 4b plots the PP of passing the hand scan depending on former hand hygiene education, interestingly showing a higher PP of passing the minimum coverage of 95% in the group of participants without former hand hygiene education.

### 3.3. Analysis of Errors in Hand Antisepsis Technique

In order to obtain a further understanding of errors in hand hygiene technique, it was analyzed which areas of the hand were most frequently affected, and this was compared between the groups.

**Figure 5:**
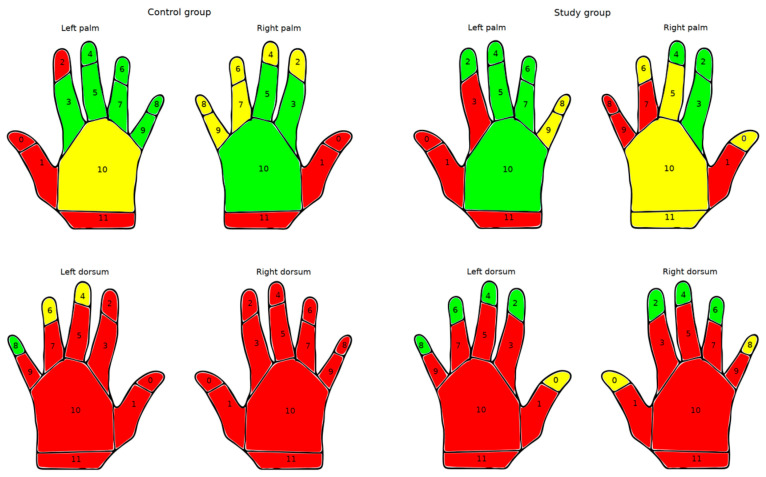
Errors in hand antisepsis technique: Distribution of errors in hand antisepsis linked to hand surface areas, each hand surface (palm and dorsum of left and right hands) divided into 12 regions (0–11). Green areas indicate that less than 5% of participants presented an error in coverage >0.5 cm^2^ in that area, yellow 5–10% of participants, and red more than 10% of participants. Left: control group, educated by an HCW; right: intervention group, educated by the video.

Figure 5 shows the detailed error rates for both the control and intervention groups for various areas of the hand surface. The data are visualized in a regional model, indicating areas where less than 5% of participants presented an error in coverage of greater than 0.5 cm^2^ in green, areas where 5–10% presented this error in yellow, and areas where more than 10% of participants presented this error in red.

Generally, both groups showed high error rates on the dorsum of the hands, on the wrists, and on the thumbs. While the intervention group demonstrated improved performance on the back of the left side compared to the control group, the failure rates on the dorsal side of the right hand and wrist were similar in both groups. Of note, the intervention group exhibited lower failure rates on the fingertips, especially on the dorsal side.

### 3.4. Evaluation of the Video-Based Approach

The video-based HH teaching approach was evaluated by the parents of the intervention group through a questionnaire and showed that the video was very well accepted. On a range from 1 (not at all comprehensible) to 5 (very comprehensible), the clarity of the video was rated as “very good” by 47 participants (94%), “good” by 2 participants (4%), and “moderate” by 1 participant. Thirty-two participants (64%) reported feeling secure (rated 5 on a scale from 1 to 5) in their ability to perform correct hand antisepsis, while eighteen participants (36%) felt relatively secure (rated 4 on a scale from 1 to 5). When asked whether they would retrospectively prefer HH education by an HCW or education through a video, 18 participants (38%) expressed a preference for personal education by an HCW, while 29 participants (62%) preferred video-based education. Three participants did not answer to this question.

The feedback provided by the Semmelweis Scanner was rated as “very helpful” (5 on a scale from 1 to 5) by 86% of the participants, “helpful” (4) by 12%, and “moderate” (3) by 2%.

In the comment section, several participants suggested making the video available to parents earlier, such as during the stay on the prepartum unit, before their baby’s admission to the NICU. Moreover, they recommended including information on areas most frequently missed during hand disinfection in the video. Four participants specifically asked for a Slovakian translation of the video.

## 4. Discussion

Hand hygiene is a crucial strategy in preventing HAIs, especially within NICUs [7]. In these settings, it is imperative that both medical staff and parents adhere rigorously to the highest standards of HH practices. However, adherence to HH protocols among parents in NICUs has been consistently reported as low in several studies [17,18,19]. This shortfall can be attributed to obstacles such as language barriers and the constrained time resources available to HCWs. Therefore, the aim of this study was to analyze the effectiveness of an educational video, available in ten different languages, combined with automated real-time feedback, in teaching parents an HH technique and to assess whether this approach is well accepted by the parents.

Our data demonstrate that the HH quality among parents educated through the video was at least as good as that observed after face-to-face education by an HCW, with the video education method actually showing a higher PP of passing the subsequent hand scan. Feedback from the parents in the intervention group was predominantly positive; the majority expressed a preference for video-based education. Therefore, the video appears to be a viable option for the HH education of parents in a NICU.

The application of technological devices for HH education is not a novel concept. A computer-assisted learning tool was shown to be at least as effective for teaching handwashing skills to nursing students as conventional face-to-face teaching methods [27]. Two previous studies have utilized videos for teaching the five moments of HH or hand washing behavior to parents, demonstrating their effectiveness in enhancing HH compliance [28,29]. However, to the best of our knowledge, no study so far has evaluated the quality of hand antisepsis using alcohol-based hand rub among parents following video-based education.

Considering the stringent requirement of a minimum 95% coverage of each hand surface to pass the scan, the PPs shown in our study for passing the initial hand scan are considered acceptable. However, we noted a significant difference in HH quality between female and male participants. Other studies have also documented differences in HH compliance and disinfectant coverage between female and male participants [30]. This discrepancy may be attributed to the fact that males have larger hands, which potentially leads to incomplete coverage of the hand surface when using the same amount of hand rub. This suggests the need for adjusted hand rub dosages [31]. Other authors debated whether male participants require different approaches compared to female participants to enhance HH compliance [32].

Interestingly, the areas of hand antisepsis most frequently missed differed between the intervention and the control groups. While both groups showed the highest failure rates on the back of the hand and the thumb, the intervention group performed significantly better on the fingertips. This distinction is particularly crucial considering the fingertips are heavily colonized areas with a high risk of cross-transmission [33]. In a recent study by Pittet et al., the sequence of the six steps of the standard World Health Organization handrub technique was altered, placing the fingertips at the first step instead of the last one. This modification resulted in a reduction in bacterial concentration on the hand surface [33]. Although our video did not adopt this novel approach, it emphasized the fingertips as critical area for hand antisepsis and visually highlighted their importance, which may have influenced the participants’ behavior. The WHO’s multimodal hand hygiene improvement strategies, along with the HH research agenda outlined by Pittet et al. in their Lancet review, underscore the significance of performance feedback as an important tool for HH enhancement [21,34]. The objective and personalized feedback provided by the Semmelweis Scanner has the potential to identify flaws in individual HH techniques and enhance hand antisepsis performance.

The advantages of the video compared to traditional face-to-face HH education include a consistent standard of HH education, time saving for HCWs, a reduction in language barriers, and the continuous availability of the HH education material. In the study group, the number of occasions parents watched the video before the first hand scan was positively associated with an increased PP of passing the subsequent hand scan. It is well established that HCWs and consequently also parents need regular reminders about the importance of HH to “keep it at the forefront of their minds” [21]. The WHO has identified “reminders” as one of the five key components of its multimodal HH improvement strategies [34]. At the NICU of the Medical University Vienna, the video is further used as a reminder, constantly displayed on a large flat screen in the parents’ waiting room, alternating with photos and videos of discharged babies to attract attention. Of note, while most studies on the HH of HCWs have not demonstrated sustained improvement in compliance following educational intervention, a study on HH compliance among parents reported a steady increase throughout the study period [29]. The authors discussed a time-limited stay in the NICU and the strong motivation of parents to do whatever is needed for their children as possible influencing factors. Interestingly, self-reported earlier HH education reduced the PP of passing the hand scan in our study. This could possibly be attributed to an overestimation of one’s own HH technique skills, though this remains speculative.

As a result of our study, we have integrated the educational video into the standard HH educational protocol for parents in a NICU at a large tertiary hospital. This protocol now includes the educational video followed by objective and individual feedback provided by the Semmelweis Scanner, with documentation in the digital patient data system. Furthermore, parents are encouraged to use the Semmelweis Scanner regularly, and the HH educational video displayed in the parents’ waiting room serves as reminder. As several participants suggested that HH education should commence before birth, we are planning to include the video in prenatal consultations.

### Limitations

Our analysis was confined to the initial hand scan conducted after HH education to evaluate hand antisepsis performance. Therefore, we were unable to assess any short- or long-term preventive effects of the video education or the performance feedback.

Moreover, we did not measure the amount of time HCWs spent on face-to-face education of parents; hence, we were not able to quantify the exact time savings attributable to the use of the educational video. However, the time required for face-to-face education varies greatly depending on individual parents, the HCW, and factors such as language barriers. Despite this variability, we can report that HCWs at the NICU where this study was conducted perceive the use of the video in combination with the Semmelweis Scanner as a significant reduction in workload, particularly on days with high admission numbers.

## 5. Conclusions

After examining whether a video-based approach is effective in teaching parents in a NICU proper HH technique, our data demonstrated the following:

- The hand hygiene quality of parents educated by video was as least as good as after individual education by a HCW;
- The majority of parents expressed a preference for video-based education;
- Participants educated by video showed better antisepsis quality for the fingertips;
- Disinfectant coverage of the hands differed between male and female participants.

In order to provide a consistent standard of HH education, we recommend the implementation of a video-based approach. This method not only saves time for HCWs but also reduces language barriers and ensures the continuous availability of HH educational materials. Additionally, the video can serve as a regular reminder of the importance and proper technique of HH. Therefore, the video-based approach appears to be a suitable and effective method for HH education for parents in the NICU.

Institutional hand hygiene is gaining more scientific recognition in the post-COVID-19 world. Whether continuous reminders of hand hygiene technique and feedback on individual hand antisepsis performance, as provided by the video-based approach used in this study, can have a positive effect on patient safety remains to be tested in future studies. Furthermore, it would be interesting to analyze whether hand dispensers with adjusted hand rub dosages can help counterbalance discrepancies in disinfectant coverage between females and males.

## Figures and Tables

**Table 1:** Demographic data and primary outcome of the participating parents.

	Total	Control Group	Intervention Group
**Demographic data**			
Participants, n	94	44	50
Male, n (%)	37 (39.4)	16 (36.4)	21 (42.0)
Age, y (%)			
18–29	25 (26.6)	11 (25.0)	14 (28.0)
30–30	56 (59.6)	25 (56.8)	31 (62.0)
40–49	11 (11.7)	6 (13.6)	5 (10.0)
50–59	2 (2.1)	2 (4.5)	0 (0.0)
Dominant hand left, n (%)	14 (14.9)	5 (11.4)	9 (18.0)
Former hand hygiene education	34 (36.2)	16 (36.4)	18 (36.0)
**Results**			
Hand scan passed, n (%)	60 (63.8)	26 (59.1)	34 (68.0)
PP passing the hand scan, % (CI)			
female		67.9 (48.9–82.4%)	75.9% (CI 57.3–88.0%)
male		43.8 (22.5–67.6%)	57.1% (36.0–76.0%)

n, number; y, years; CI, confidence interval.

## Data Availability

All data requests should be submitted to the corresponding author for consideration. Access to anonymized data may be granted following review.

## Abbreviations

CI, confidence interval; HAI, healthcare-associated infection; HCW, healthcare worker; HH, hand hygiene; NICU, neonatal intensive care unit; PP, predicted probability; WHO, world health organization.

## References

[1] Legeay C., Bourigault C., Lepelletier D., Zahar J.R. (2015). Prevention of healthcare-associated infections in neonates: Room for improvement. J. Hosp. Infect..

[2] Fleiss N., Tarun S., Polin R.A. (2022). Infection prevention for extremely low birth weight infants in the NICU. Semin. Fetal Neonatal Med..

[3] Giannoni E., Agyeman P.K.A., Stocker M., Posfay-Barbe K.M., Heininger U., Spycher B.D., Bernhard-Stirnemann S., Niederer-Loher A., Kahlert C.R., Donas A. (2018). Neonatal Sepsis of Early Onset, and Hospital-Acquired and Community-Acquired Late Onset: A Prospective Population-Based Cohort Study. J. Pediatr..

[4] Flannery D.D., Edwards E.M., Coggins S.A., Horbar J.D., Puopolo K.M. (2022). Late-Onset Sepsis Among Very Preterm Infants. Pediatrics.

[5] Baron R., Taye M., der Vaart I.B., Ujčič-Voortman J., Szajewska H., Seidell J.C., Verhoeff A. (2020). The relationship of prenatal antibiotic exposure and infant antibiotic administration with childhood allergies: A systematic review. BMC Pediatr..

[6] Rasmussen S.H., Shrestha S., Bjerregaard L.G., Ängquist L.H., Baker J.L., Jess T., Allin K.H. (2018). Antibiotic exposure in early life and childhood overweight and obesity: A systematic review and meta-analysis. Diabetes Obes. Metab..

[7] Pittet D., Allegranzi B., Boyce J. (2009). The World Health Organization Guidelines on Hand Hygiene in Health Care and their consensus recommendations. Infect. Control Hosp. Epidemiol..

[8] Pittet D., Allegranzi B., Sax H., Dharan S., Pessoa-Silva C.L., Donaldson L., Boyce J.M. (2006). Evidence-based model for hand transmission during patient care and the role of improved practices. Lancet Infect. Dis..

[9] McGuckin M., Govednik J. (2013). Patient empowerment and hand hygiene, 1997–2012. J. Hosp. Infect..

[10] Ramezani T., Hadian Shirazi Z., Sabet Sarvestani R., Moattari M. (2014). Family-centered care in neonatal intensive care unit: A concept analysis. Int. J. Community Based Nurs. Midwifery.

[11] Mehler K., Hucklenbruch-Rother E., Trautmann-Villalba P., Becker I., Roth B., Kribs A. (2020). Delivery room skin-to-skin contact for preterm infants-A randomized clinical trial. Acta Paediatr..

[12] Arya S., Naburi H., Kawaza K., Newton S., Anyabolu C.H., Bergman N., Rao S.P.N., Mittal P., Assenga E., Gadama L. (2021). Immediate “Kangaroo Mother Care” and Survival of Infants with Low Birth Weight. N. Engl. J. Med..

[13] Boundy E.O., Dastjerdi R., Spiegelman D., Fawzi W.W., Missmer S.A., Lieberman E., Kajeepeta S., Wall S., Chan G.J. (2016). Kangaroo Mother Care and Neonatal Outcomes: A Meta-analysis. Pediatrics.

[14] Musu M., Lai A., Mereu N.M., Galletta M., Campagna M., Tidore M., Piazza M.F., Spada L., Massidda M.V., Colombo S. (2017). Assessing hand hygiene compliance among healthcare workers in six Intensive Care Units. J. Prev. Med. Hyg..

[15] Rittenschober-Böhm J., Bibl K., Schneider M., Klasinc R., Szerémy P., Haidegger T., Ferenci T., Mayr M., Berger A., Assadian O. (2020). The association between shift patterns and the quality of hand antisepsis in a neonatal intensive care unit: An observational study. Int. J. Nurs. Stud..

[16] Lambe K.A., Lydon S., Madden C., Vellinga A., Hehir A., Walsh M., O’Connor P. (2019). Hand Hygiene Compliance in the ICU: A Systematic Review. Crit. Care Med..

[17] Chandonnet C.J., Boutwell K.M., Spigel N., Carter J., DeGrazia M., Ozonoff A., Flaherty K. (2017). It’s in Your Hands: An Educational Initiative to Improve Parent/Family Hand Hygiene Compliance. Dimens. Crit. Care Nurs..

[18] Maria A., Sooden A., Wadhwa R., Kaur R., Gaur I., Lhamo K., Nagaratna V. (2022). Improving handwashing among parent-attendants visiting a newborn unit practising family participatory care. BMJ Open Qual..

[19] Randle J., Firth J., Vaughan N. (2013). An observational study of hand hygiene compliance in paediatric wards. J. Clin. Nurs..

[20] Kletečka-Pulker M., Parrag S., Doppler K., Völkl-Kernstock S., Wagner M., Wenzel T. (2021). Enhancing patient safety through the quality assured use of a low-tech video interpreting system to overcome language barriers in healthcare settings. Wien. Klin. Wochenschr..

[21] Lotfinejad N., Peters A., Tartari E., Fankhauser-Rodriguez C., Pires D., Pittet D. (2021). Hand hygiene in health care: 20 years of ongoing advances and perspectives. Lancet Infect. Dis..

[22] Tartari E., Bellissimo-Rodrigues F., Pires D., Fankhauser C., Lotfinejad N., Saito H., Suchomel M., Kramer A., Allegranzi B., Boyce J. (2024). Updates and future directions regarding hand hygiene in the healthcare setting: Insights from the 3rd ICPIC alcohol-based handrub (ABHR) task force. Antimicrob. Resist. Infect. Control.

[23] Haidegger T., Nagy M., Lehotsky A., Szilagyi L. (2011). Digital imaging for the education of proper surgical hand disinfection. Med. Image Comput. Comput. Assist. Interv..

[24] Lehotsky A., Szilagyi L., Bansaghi S., Szeremy P., Weber G., Haidegger T. (2017). Towards objective hand hygiene technique assessment: Validation of the ultraviolet-dye-based hand-rubbing quality assessment procedure. J. Hosp. Infect..

[25] Patientensicherheit in der Neonatologie—Händedesinfektion. https://www.youtube.com/watch?v=d_mUWk20_u0.

[26] Nagy K., Lehotsky A., Bansaghi S., Haidegger T. (2017). Identyfining optimal pass-fail criterion for hand hygiene technique_Meeting abstracts from International Conference on Prevention & Infection Control (ICPIC 2017). Antimicrob. Resist. Infect. Control.

[27] Bloomfield J., Roberts J., While A. (2010). The effect of computer-assisted learning versus conventional teaching methods on the acquisition and retention of handwashing theory and skills in pre-qualification nursing students: A randomised controlled trial. Int. J. Nurs. Stud..

[28] Lary D., Calvert A., Nerlich B., Segal J., Vaughan N., Randle J., Hardie K.R. (2020). Improving children’s and their visitors’ hand hygiene compliance. J. Infect. Prev..

[29] Chen Y.C., Chiang L.C. (2007). Effectiveness of hand-washing teaching programs for families of children in paediatric intensive care units. J. Clin. Nurs..

[30] Szilagyi L., Haidegger T., Lehotsky A., Nagy M., Csonka E.A., Sun X., Ooi K.L., Fisher D. (2013). A large-scale assessment of hand hygiene quality and the effectiveness of the “WHO 6-steps”. BMC Infect. Dis..

[31] Price L., Gozdzielewska L., Alejandre J.C., Jorgenson A., Stewart E., Pittet D., Reilly J. (2022). Systematic review on factors influencing the effectiveness of alcohol-based hand rubbing in healthcare. Antimicrob. Resist. Infect. Control.

[32] Seliger G., Krol I., Worlitzsch D., Kantelhardt E.J., Moritz S., Tchirikov M. (2020). Reduction of Visitor- and Staff-Associated Risk of Infection by Complex Intervention in the Department of Feto-Maternal Medicine. Z. Geburtshilfe Neonatol..

[33] Pires D., Bellissimo-Rodrigues F., Soule H., Gayet-Ageron A., Pittet D. (2017). Revisiting the WHO “How to Handrub” Hand Hygiene Technique: Fingertips First?. Infect. Control Hosp. Epidemiol..

[34] WHO (2009). WHO Guidelines Approved by the Guidelines Review Committee. WHO Guidelines on Hand Hygiene in Health Care: First Global Patient Safety Challenge Clean Care Is Safer Care.

